# Oxidative stress-mediated beta cell death and dysfunction as a target for diabetes management

**DOI:** 10.3389/fendo.2022.1006376

**Published:** 2022-09-23

**Authors:** Svetlana Dinić, Jelena Arambašić Jovanović, Aleksandra Uskoković, Mirjana Mihailović, Nevena Grdović, Anja Tolić, Jovana Rajić, Marija Đorđević, Melita Vidaković

**Affiliations:** Department of Molecular Biology, Institute for Biological Research “Siniša Stanković” - National Institute of Republic of Serbia, University of Belgrade, Belgrade, Serbia

**Keywords:** oxidative stress, pancreatic beta cells, diabetes, epigenetics, CRISPR-Cas9, diabetes management

## Abstract

The biggest drawback of a current diabetes therapy is the treatment of the consequences not the cause of the disease. Regardless of the diabetes type, preservation and recovery of functional pancreatic beta cells stands as the biggest challenge in the treatment of diabetes. Free radicals and oxidative stress are among the major mediators of autoimmune destruction of beta cells in type 1 diabetes (T1D) or beta cell malfunction and death provoked by glucotoxicity and insulin resistance in type 2 diabetes (T2D). Additionally, oxidative stress reduces functionality of beta cells in T2D by stimulating their de-/trans-differentiation through the loss of transcription factors critical for beta cell development, maturity and regeneration. This review summarizes up to date clarified redox-related mechanisms involved in regulating beta cell identity and death, underlining similarities and differences between T1D and T2D. The protective effects of natural antioxidants on the oxidative stress-induced beta cell failure were also discussed. Considering that oxidative stress affects epigenetic regulatory mechanisms involved in the regulation of pancreatic beta cell survival and insulin secretion, this review highlighted huge potential of epigenetic therapy. Special attention was paid on application of the state-of-the-art CRISPR/Cas9 technology, based on targeted epigenome editing with the purpose of changing the differentiation state of different cell types, making them insulin-producing with ability to attenuate diabetes. Clarification of the above-mentioned mechanisms could provide better insight into diabetes etiology and pathogenesis, which would allow development of novel, potentially more efficient therapeutic strategies for the prevention or reversion of beta cell loss.

## Introduction

Human pancreas is a complex gland with dual exocrine and endocrine function. The endocrine part of the pancreas is represented by Langerhans islets scattered throughout the exocrine pancreas, making up only 1-5% of the mass of the gland (1). Islets poses mantle-core architecture so that the core is mainly made up of insulin-secreting beta cells (70-80%) surrounded by a discontinuous mantle of glucagon-secreting alpha/polypeptide-secreting PP cells (15-20%) and somatostatin-secreting delta cells (5%) (2). Insulin is involved in the regulation of various physiological processes and plays central role in glucose homeostasis. Rise in circulating glucose concentration after meal induces the secretion of insulin which enhances glucose uptake and glycogen synthesis in the liver and muscles, intensifies production of triglycerides which are stored in adipocytes and stimulates protein synthesis. Additionally, insulin inhibits secretion of glucagon and thereby reduces glucose output in the liver (3). Any deficit of insulin or a decreased tissue response to insulin action impairs glucose, lipid and protein metabolism and leads to chronic hyperglycemia and diabetes. Based on the pathophysiological processes that underlie the development of disease several basic categories of diabetes are distinguished. Type 1 diabetes (T1D) affects about 10% of diabetic patients most commonly children and adolescents and occurs as a consequence of selective autoimmune destruction of pancreatic beta cells (4). At the time of diagnosis, reduction of 70-90% of beta cell mass as well as increase in the circulation of insulin autoantibodies (IAA), glutamic acid decarboxylase autoantibodies (GADA), insulinoma 2-associated autoantibodies (IA-2 and IA-2β) and islet-cell cytoplasmic autoantibodies (ICA) are detected in 60-80% of diabetic patients (5). Type 2 diabetes (T2D) is the most common (85-90%) form of diabetes and usually occurs in patients over the age of forty, but it is increasingly being detected in a younger people and adolescents (6). T2D is characterized by insulin resistance, disturbances in the insulin secretion and progressive loss of 25-50% of pancreatic beta cells in the course of diabetes (7). Insulin deficiency and long-term hyperglycemia leads to severe diabetic complications such as nephropathy, retinopathy, neuropathy, cardiovascular and liver disorders and consequently to premature death (8).

The high frequency and prevalence of the disease makes diabetes one of the four most common non-communicable diseases. According to the International Diabetes Federation (IDF) (4), in 2021 there were about 537 million adults (20-79 years) worldwide with diabetes, of which almost 50% were undiagnosed. More than 1.2 million children and adolescents (0-19 years) are living with T1D, while 541 million adults are at increased risk of developing T2D. It is estimated that by 2045, the number of people suffering from diabetes in the world will increase to 783 million, with the largest increase in the number of patients expected in developing countries. A large number of people with diabetes require significant costs for therapy, so that over 966 billion dollars are spent annually for diabetes treatment. Notwithstanding, diabetes therapy does not give desired results and nearly four million diabetic patients die each year worldwide. Pandemic scale of diabetes requires the improvement of current therapy, and it is a prerequisite to understand in detail pathogenesis of the disease.

Numerous studies dedicated to a clarification of the mechanisms involved in beta cell dysfunction revealed that excess of reactive oxygen and nitrogen species (ROS and RNS, respectively) and resulting non-physiological oxidative stress mediate various pathophysiological processes that underlie beta cell malfunction, dedifferentiation/transdifferentiation and death (9–14). ROS such as superoxide anion ( $\cdot {\text{O}}_{2}^{-}$ ), hydrogen peroxide (H_2_O_2_), peroxyl radical (ROO^•^), hydroxyl radical (HO^•^) and RNS such as nitric oxide (NO^•^), nitrogen dioxide ( $\cdot {\text{NO}}_{2}^{-}$ , ${\text{NO}}_{2}^{-}$ , nitrite ion) and peroxynitrite anion (ONOO^-^) are products of normal cellular metabolism and depending on their concentration can have beneficial or harmful effects (15). Under physiological conditions, ROS and RNS molecules are present in low concentrations and are involved in the regulation of signaling processes, differentiation, cell growth and migration, gene expression, posttranslational modifications and cellular defense against various infectious agents (16). New insights indicate that redox-related signaling requires continuous control of the steady-state redox set point by compartmentalized generation and removal of reactive species in order to preserve physiologically relevant oxidative stress denoted as oxidative eustress crucial for various biological processes and lifespan (17). However, in large quantities, either due to excessive production or due to disruption of the antioxidant system, reactive molecules initiate cell damage through lipid peroxidation, protein oxidation and DNA damage (18, 19). In addition, ROS and RNS indirectly cause oxidative tissue damage by activating numerous cell signaling pathways. The level of reactive species is regulated by a complex system of antioxidant protection including endogenous and exogenous antioxidants that prevent, delay or remove oxidative damage from target molecules (20). There is no universal “best” antioxidant and different antioxidants react with different free radicals with varying efficiency to protect target molecules. This review summarizes up to date clarified redox-related mechanisms involved in beta cell identity and death and discusses the potential of natural antioxidants in improving antioxidant capacity and function of beta cells in diabetes.

Growing body of evidence indicate that epigenetic mechanisms such as DNA methylation, chromatin architectural modification and non-coding RNA determine identity of beta cells during embryogenesis and postnatal maturation and maintain their functioning in homeostasis (21). Diabetes progression and oxidative stress disarrange those epigenetic signatures causing dysfunction and loss of beta cells. Capacity of antioxidant compounds to improve viability of beta cells and reduce diabetic complications by modulating specific epigenetic markers (22–24) opens the avenue for defining specific epigenetic targets in the treatment of diabetes by antioxidants. Therefore, this review provides an overview of the studies supporting this point. In addition, by summarizing recent findings regarding the application of the state-of-the-art CRISPR/Cas9 technology in diabetes, this review emphasis a huge potential of targeted epigenome editing in changing the differentiation state of different cell types into insulin-producing cells with ability to attenuate diabetes. Complete understanding of the mechanisms underlying decreased tissue response to insulin action, as well as of the processes being crucially implicated in the preservation and recovery of beta cell mass would make diabetes no longer a chronic but a curable disease.

## Redox-related mechanisms involved in beta cell death and dysfunction

**Figure 1:**
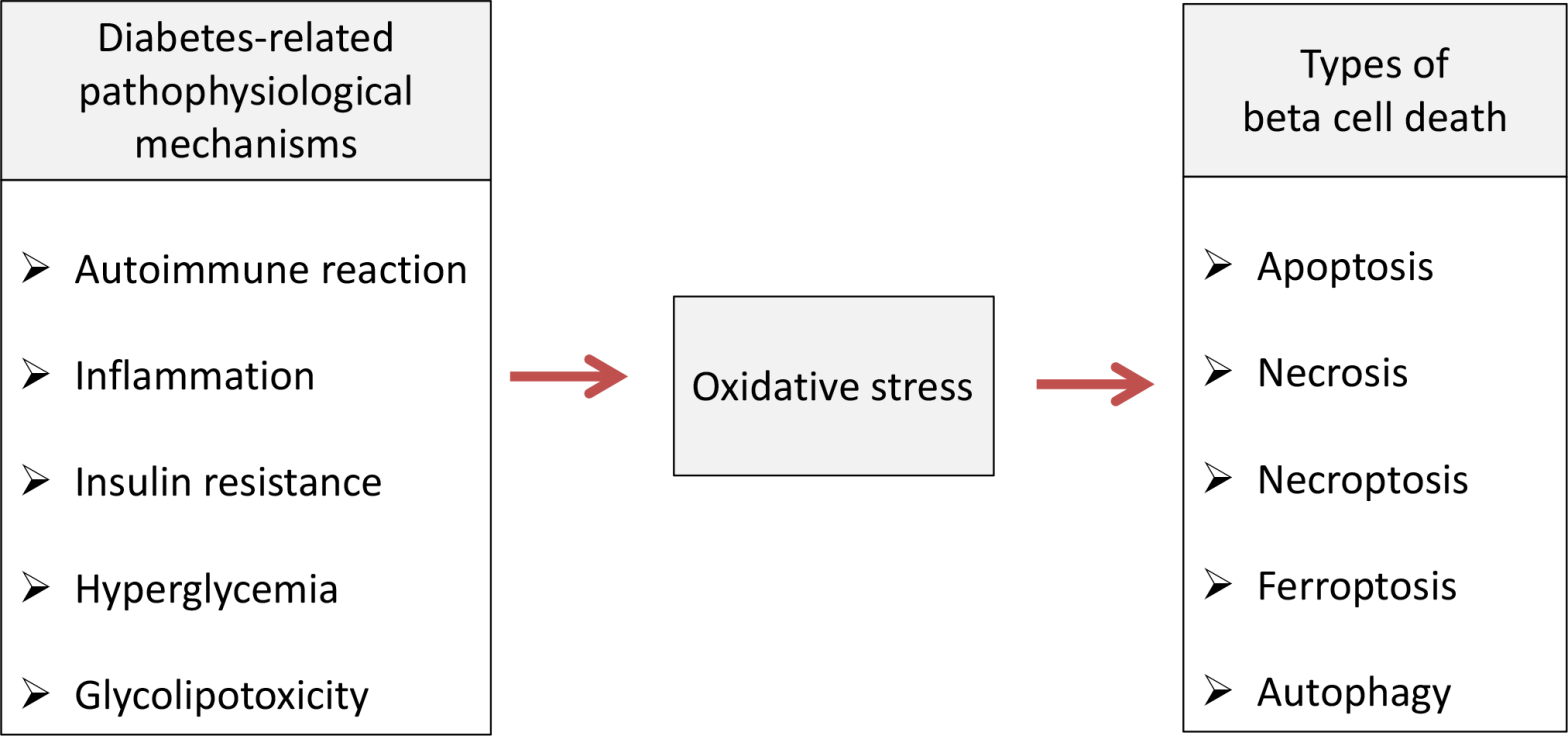
Oxidative stress-mediated pathophysiological mechanisms cause various types of beta cell death in diabetic condition. Pathophysiological processes such as autoimmune reaction, inflammation, insulin resistance, hyperglycemia/glucotoxicity and lipotoxicity lead to pancreatic beta cell death in diabetic condition by inducing oxidative stress which is involved in apoptosis, necrosis, necroptosis, ferroptosis and autophagy.

Pancreatic beta cell failure occurs as the final step in T1D and T2D due to activation of various pathophysiological mechanisms such as autoimmune reaction, inflammation, insulin resistance, hyperglycemia/glucotoxicity, lipotoxicity and de-/trans-differentiation (25). Numerous clinical and experimental studies revealed that non-physiological oxidative stress represents underlying factor through which those mechanisms disrupt pancreatic beta cells structure and function and lead to development of diabetic complications (15, 26). The role of oxidative stress in mediating various types of beta cell death is summarized on **Figure 1**.

In beta cells, oxidative stress is involved in the reduction of the activity of key regulators of insulin expression and other beta cell specific genes (11, 27). The adverse effects of oxidative stress on beta cells are pronounced in comparison to other cell types due to a low level of antioxidant capacity of beta cells. Comparative analysis of the gene expression of antioxidative enzymes in different cell types of islets prepared from human pancreatic sections showed lower expression of $\cdot {\text{O}}_{2}^{-}$ eliminating superoxide dismutases CuZn/Mn-SOD (1.4-fold) as well as of H_2_O_2_ eliminating glutathione peroxidase (GPx1) (3-fold) and catalase (CAT) (15-fold) in beta than in non-beta cells (28). Recent findings suggest that beta cells possess capacity to fight against micromolar levels of H_2_O_2_ through a thioredoxin/reductase-dependent mechanism thus not being as sensitive to oxidative damage as previously thought (29, 30). This presumption is based on demonstrated ability of human EndoC-βH1 beta cell line (similar to primary human beta cells) to detoxify continuously generated physiological level (50 µM) of H_2_O_2_ for 4 h through the peroxiredoxin/thioredoxin antioxidant system. However, in the same study, 100 µM H_2_O_2_ applied as a bolus to EndoC-βH1 beta cells reduced cell viability and induced DNA damage along with the depletion of cellular energy (29). Given these data, concerns remain as to whether peroxiredoxin/thioredoxin system would be effective in beta cell protection after the prolonged exposure to physiologically relevant H_2_O_2_ concentration (31). On the other hand, poor antioxidant capacity of beta cells make them susceptible to ROS mediated signaling involved in the regulation of glucose-stimulated insulin secretion (GSIS) in physiological conditions (32). Reactive species are mainly produced in mitochondrial respiratory chain that provides adenosine triphosphate (ATP) for GSIS under increased glycolytic and tricarboxylic acid (TCA) flux (27). ROS were shown to stimulate activation of ryanodine receptors (RyR) required for the increase of intracellular free Ca^2+^ in the course of GSIS in rat islet beta cells (33). However, continued increase in glycolytic flux due to impaired glucose clearance in diabetes pathogenesis results in ROS/RNS overproduction in beta cells with potential pathological consequences (27). Elevated levels of ROS reduce expression and secretion of insulin and lead to beta cell damage, suggesting critical role of redox balance and preservation of oxidative eustress (17) for a proper beta cell functioning (34).

### Autoimmune destruction of beta cells and inflammation

Though it has long been thought that the autoimmune destruction of beta cells characterizes only T1D, the role of the autoimmune reaction as additional pathological mechanism of selective beta cells disruption in T2D is increasingly being recognized. In T1D the autoimmune reaction is triggered in genetically predisposed persons in response to not entirely clarified environmental factors, while in the case of T2D the trigger for autoimmune destruction of beta cells is chronic inflammation due to obesity and metabolic disorders (35). Prolonged hyperglycemia is associated with sustained oxidative stress which triggers the expression of different pathogenic proinflammatory pathways and activates inflammation-regulated genes (36). The autoimmune process in pancreatic beta cells is characterized by infiltration of macrophages, dendritic and T cells in the islets of Langerhans. Proinflammatory cytokines such as interferon-gamma (IFN)-γ, tumor necrosis factor-alpha (TNF-α) and interleukin 1-beta (IL-1β) and reactive molecules such as nitric oxide (NO) and various related free-radical and oxidant species produced by infiltrated immune cells are major mediators of the induction of apoptosis, the main form of cell death observed through biopsies of islets of rats and humans with T1D (37). Oxidative stress activates mitochondrial or intrinsic pathway of apoptotic beta cell death mediated by family of Bcl-2 proteins (38, 39), while interaction of apoptosis stimulating fragment ligand (FasL) and TNF-α with corresponding receptors on beta cell surface initiate the extrinsic pathway of apoptotic cell death (40). Resulting death signals recruit procaspases and promote their activation into caspases (cysteine-aspartic proteases) leading to disruption of beta cells (41). Activated IL-1β has a crucial role in the amplification and support of FasL and NO effects in beta cell dysfunction and death (42). Through defined chain of events, interaction of IL-1β with IL-1R1 receptor on the surface of beta cells results with an activation of nuclear factor kappa B protein (NF-κB) which reduces activity of pancreatic and duodenal homeobox factor-1 (Pdx1) transcription factor crucial for beta cell functioning and induces inducible nitric oxide synthase (iNOS) expression and consequently NO generation (43). In accordance, activated iNOS and ${\text{ONO}}_{2}^{-}$ were detected in pancreas of non-obese diabetic (NOD) mice, whereas iNOS-deficient mice are protected from streptozotocin (STZ) induced diabetes (44). Formation of ${\text{ONO}}_{2}^{-}$ promotes the release of cytochrome c from the mitochondrial membrane which mediates activation of executioner caspase leading to apoptosis (45). NO also provokes fragmentation of DNA, reacts with prosthetic groups of transcription factors required for their biological function and inhibits enzymatic activity required for synthesis of ATP and insulin (46).

Secretion of IL-1β and TNF-α in macrophage and dendritic cells is further stimulated by IFN-γ. Interaction of TNF-α with its TNFR-1 receptor expressed on beta cells triggers signaling that leads to the activation of caspases 3, 6 and 7 (37). Besides inducing apoptotic cell death, activation of TNF-α/TNFR-1 signaling promotes beta cell death by programmed necrosis i.e. necroptosis, executed through excessive ROS production and fragmentation of DNA (47). ROS are produced through Fenton reaction due to TNF-α-stimulated degradation of ferritin and accumulation of iron ions Fe^2+^ and Fe^3+^, as well as through increased influx of α-ketoglutarate to Krebs cycle and stimulated reduction-oxidation reactions in the respiratory chain (37). While exposure of RINm5f cells to IL-1β promoted iNOS-mediated NO**^•^** production, combination of IL-1 β, TNF-α and INF- γ induced accumulation of H_2_O_2_ and production of highly toxic HO**^•^** (48). Excessive level of HO**^•^** induces lipid peroxidation of cell membranes and stimulates apoptosis-inducing factor (AIF) translocation from mitochondria to nucleus where it induces large-scaled DNA fragmentation and necroptosis (49).

### Insulin resistance and glucotoxic induced beta cell death

Hyperglycemia, insulin resistance and high insulin requirements in T2D cause compensatory hyperinsulinemia and expansion of beta cells which gradually leads to a loss of beta cell mass (50). Under normoglycemic conditions, glucose metabolites are mostly subjected to oxidative phosphorylation, while hyperglycemia promotes additional biosynthetic pathways including glucose autooxidation as the main source of ROS production in diabetic condition (51). In combination with transition metals, glucose oxidation generates ${\text{O}}_{2}^{-}$ and subsequently H_2_O_2_ leading to a production of extremely reactive HO^-^. In addition, hyperglycemia activates NF-κB and increases the expression of NAD(P)H oxidase (NOX) and iNOS, resulting with $\cdot {\text{O}}_{2}^{-}$ and NO^•^ production and ${\text{ONO}}_{2}^{-}$ formation (52). Peroxynitrite induces damage of DNA by introducing single-strand breaks which is a stimulus for the activation of poly (ADP-ribose) polymerase 1 (PARP-1) (53). PARP-1 binds to damaged DNA and catalyzes cleavage of NAD^+^ to nicotinamide and ADP-ribose which is further used for poly(ADP-ribose) polymers formation and their covalent binding to proteins (44). PolyADPribosylation of glyceraldehyde-3-phosphate dehydrogenase (GAPDH) reduces its activity (54). Accumulation of GAPDH upstream glycolytic intermediates such as glucose, glucose-6-phosphate, fructose-6-phosphate, fructose-1,6- diphosphate, glyceraldehyde-3-phosphate and dihydroxyacetone phosphate causes enhanced activity of sorbitol and hexosamine metabolism, protein kinase C activation, enediol formation, polyol pathway and glycation, all leading to ROS overproduction (55). In addition, activation of polyol pathway and aldose reductase enzyme activity reduces concentration of nicotinamide adenine dinucleotide phosphate (NADPH) leading to reduction of antioxidant mechanisms in beta cells (37). Hyperglycemia-associated ROS overproduction promotes an increase of IL-1β expression in beta cells which mediates programmed cell death known as pyroptosis, managed through activation of caspase 1 and formation of inflammasome complex (56, 57). Prolonged hyperglycemia also stimulates inflammatory processes and proapoptotic mechanisms mediated by generation of NO and NO-related species. IL-1β-stimulated iNOS expression and NF-κB activation in turn induces iNOS and IL-1β autostimulation, thus promoting beta cell dysfunction (58).

Cell models for insulin resistance such as dexamethasone- and TNF-α-treated adipocytes revealed that ROS overproduction may be a key trait of insulin resistance (59). The oxidation rate of the redox-sensitive dye dichlorofluorescein (DCF) was higher by 65% in dexamethasone-treated and by 50% in TNF-α-treated insulin resistant cells. Elevated level of protein carbonylation in dexamethasone-treated (by 110%) and in TNF-α-treated (by 50%) adipocytes reflected cumulative oxidative stress in insulin-resistant cells and pointed that the increase in ROS level precedes and triggers the onset of detectable insulin resistance. It must be emphasized that insulin resistance exhausts beta cells through high insulin demand and compensatory hyperinsulinemia eventually affecting their survival (55). Insulin resistance in T2D is often accompanied by pathogenic effects of elevated levels of plasma fatty acids. Since a persistently raised glucose concentration precedes diabetes-related lipotoxicity, the term glucolipotoxicity is used for describing deleterious action of lipids on beta cells (55). Oleate-treated MIN6 cells exhibited increased level of H_2_O_2_, while exposure of INS-1E cells to palmitate promoted $\cdot {\text{O}}_{2}^{-}$ formation in mitochondria (60, 61). Treatment of isolated islets and beta cells with elevated levels of fatty acids results with oxidative stress-related impaired insulin expression, inhibition of GSIS and induction of apoptotic cell death (62–65). Experimental and clinical studies indicate increased levels of oxidative stress parameters in circulation, oxidative tissue damage and a positive correlation between oxidative markers and impaired GSIS in islets in diabetic condition (66). Increased levels of oxidative stress markers such as hydroperoxides, 8-hydroxy-guanine, 8-epi-PGF2α and DNA base oxidation were detected in circulation of diabetic patients (67, 68). Exposure of human islets to high glucose concentration or rat islets to excess levels of glucose metabolite D-glyceraldehyde was accompanied by ROS overproduction and the decrease of insulin content and GSIS (69, 70). Concentrations of nitrotyrosine and 8-Hydroxydeoxyguanosine (8-OHdG) oxidative stress markers were significantly increased in pancreatic islets isolated from T2D cadaveric organ donors and they were correlated with impairment of insulin expression and GSIS (71).

High glucose level is accompanied by iron overload which leads to insulin resistance and beta cell injury by iron-related programmed cell death termed ferroptosis (72, 73). Mainly characterized by the shrinkage of mitochondria and by lipid peroxidation, ferroptosis is mediated by glutathione (GSH)/glutathione peroxidase 4 (Gpx4) pathway (74). Therefore, GSH deficiency and inhibition of GSH synthesis could provoke ferroptosis, while lipophilic-/iron-chelating agents and antioxidants can prevent this type of cell death (75). Recently, Stančić and coworkers (76) analyzed the ability of diabetogenic agents such as high glucose (HG), proinflammatory cytokines, H_2_O_2_ and STZ to induce ferroptosis in Rin-5F beta cell line *in vitro*, and have analyzed the share of ferroptosis in beta cell damage *in vivo* using STZ-diabetic mice. All tested *in vitro* treatments decreased viability of Rin-5F cells which was associated with elevated levels of iron, ROS, lipid peroxides, inactivation of NF-E2-related factor 2 (Nrf-2) along with decreased mitochondrial membrane potential (MMP) and expression of Gpx4. Those effects were diminished in HG, H_2_O_2_ and STZ treatments of beta cells by ferroptosis inhibitor ferrostatin 1 (Fer-1) which failed to improve MMP and cell viability in cytokines-treated Rin-5F cells. Moreover, Fer-1 increased the number of insulin-positive cells in islets of STZ-diabetic mice by decreasing macrophage infiltration and accumulation of lipid peroxides. Those findings strongly suggest that high glucose level and ROS overproduction, but not proinflammatory cytokines, induce loss of beta cells by ferroptosis under diabetic condition (76).

Growing body of evidence indicate association of defects in autophagy with insulin resistance, obesity and beta cell dysfunction in diabetes (77, 78). This lysosomal degradation machinery includes macroautophagy, crinophagy and microautophagy which regulate cellular homeostasis by recycling damaged macromolecules and organelles for new protein synthesis and energy production (78). Dysfunctional autophagy in beta cells under oxidative stress conditions could result with accumulation of excess proteins and their toxic effects (71). Hence, rise of autophagy in islets of diabetic db/db and high-fat diet (HFD) fed C57BL/6 mice suggest its role in adaptive response of beta cells to fight against insulin resistance and insulin deficiency (79). Autophagy may also protect cells from H_2_O_2_-induced single- or double-strand DNA breaks and deleterious effects of increased activity of PARP-1 (80). Activation of PARP-1 and subsequent intracellular NAD^+^ and ATP depletion leads to inhibition of proinsulin synthesis and increases AIF translocation from mitochondria to the nucleus, resulting with chromatin condensation, DNA fragmentation and programmed necrotic cell death independent of the caspase cascade (44, 81). On the other hand, Huang et al. (80) showed that PARP-1 could stimulate autophagy by promoting serine/threonine protein kinase LKB1-AMP-activated protein kinase (AMPK)–mammalian target of rapamycin (mTOR) pathway, suggesting that beta cell survival depends on the balance between necrosis and autophagy mediated by two particular PARP-1-pathways. Oxidative stress and DNA damage are among multiple cellular stressors that stimulate autophagy of beta cells (82). ROS promotes autophagy by several mechanisms including activation of mitogen-activated protein kinases (MAPKs) such as c-Jun NH2-terminal kinase 1 (JNK1) which inhibits insulin receptor substrate 1 (IRS1) (83). Hydrogen peroxide may stimulate PKR-like eIF2α kinase (PERK) which, by phosphorylation of general autophagy regulator initiation factor 2α (eIF2α), stimulates expression of LC3 necessary for sustained autophagy (84). By reducing IkBα translation, PERK also activates autophagy contributing factor NF-kB (85). Hydrogen peroxide directly oxidizes Atg4 proteases and enhances their activity toward accelerated formation of proteolytically mature LC3 (86) **Figure 1**.

### Beta cell identity and de-/trans-differentiation

Generally accepted view that diabetes-associated beta cell loss is primarily caused by beta cell death was recently challenged. It is increasingly being recognized that loss of beta cell identity through de- and trans-differentiation processes contribute to failure of beta cells (50). Rodent based *in vitro* and *in vivo* studies indicate that preservation of beta cell identity is regulated by several transcription factors identified as critical for development, maturity, regeneration and functioning of beta cells. Dedifferentiation is characterized by decreased expression of key beta cell markers such as Pdx-1, musculoaponeurotic fibrosarcoma oncogene homolog A (MafA), homeobox protein (Nkx6.1), neurogenic differentiation factor 1 (NeuroD1) and forkhead box protein O1 (Foxo1) along with increased expression of beta cell “disallowed” genes such as progenitor Neurogenin3, SRY-box transcription factor 9 (SOX9) or monocarboxylate transporter MCT1 and lactate dehydrogenase genes (87). On the other hand, transdifferentiation implies transition of beta cells into other types of hormone producing islet cells. Mice lacking Foxo1 transcription factor in beta cells exhibited reduction of beta cell mass due to dedifferentiation of beta cells into Neurogenin3-, Nanog-, octamer-binding transcription factor 4 (Oct4-) and cell cycle regulator L-Myc-expressing progenitor-like cells and alpha-like cells, resulting with hyperglycemia and hyperglucagonemia (88). Likewise, independent studies provided evidence that dedifferentiation may also occur in human beta cells through loss of transcription factors necessary for maintaining mature beta cell identity. Namely, selective loss of Pdx1, MafA and Nkx6.1 markers was detected in pancreatic islets from T2D cadaveric organ donors which was associated with a marked insulin reduction (89, 90). It can be assumed that dedifferentiation represents a defense mechanism by which beta cells avoid the autoimmune reaction (10). This is supported by data showing that loss of beta cell maturity genes and acceptance of stemness-like proprerties of beta cells repress the autoimmune attack and development of diabetes in NOD mice (91).

Deletion of beta cell specific Pdx-1 or overexpression of alpha cell specific aristaless-related homeobox gene (Arx) transcription factor leads to decreased number of beta cells and increased number of alpha cells in rodents (92). Similar change in beta cell-fate was detected in diabetic db/db mice characterized by loss of insulin as well as of Pdx-1 and MafA factors (88). Pdx-1 plays an important role in pancreas development and differentiation, as well as in maintaining normal beta cell function, while MafA transcription factor primarily regulates insulin gene expression but is also involved in development and proliferation of beta cells (11, 27). Targeted removal of Pdx1 from beta cells led to severe hyperglycemia in PKO mice associated with reprogramming of majority of Pdx1-deleted beta cells into alpha-like cells including de-repression of MafB transcription factor specific for alpha cells (12). These data suggest that Pdx1 plays an essential role in regulating beta cell identity by concurrently activating beta cell genes and repressing genes associated with alpha cell fate. The lack of NKX6.1 in beta cells promotes gaining of delta-cell phenotype (93), while deficiency of PAX6 or NKX2.2 establishes epsilon-like or polyhormonal-cells, respectively (13, 14).

**Figure 2:**
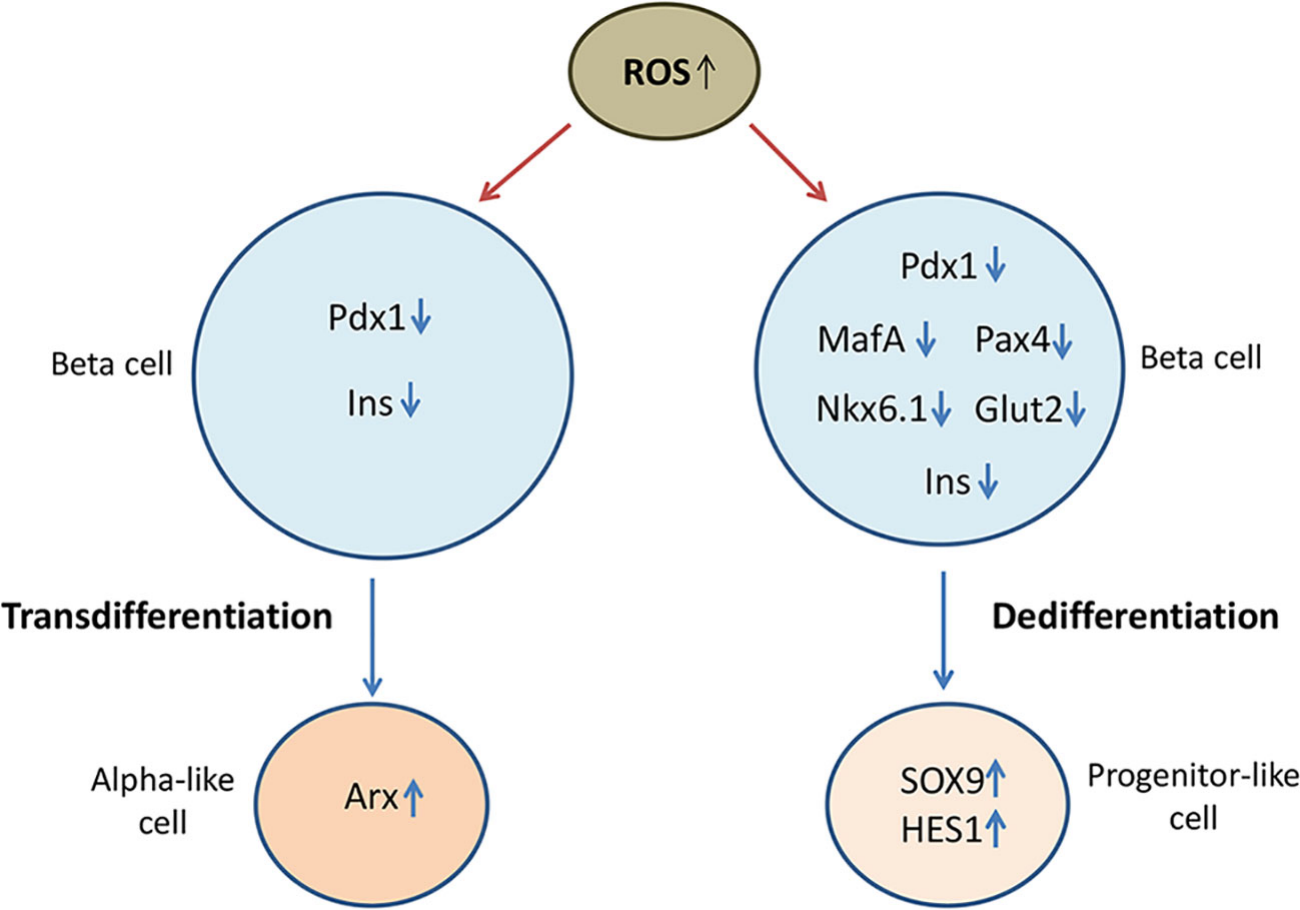
Oxidative stress compromises beta cell identity by altering expression of beta cell maturity genes and beta cell disallowed genes. Loss of beta cell identity through de-/trans-differentiation processes contributes to beta cell failure in diabetic condition. Oxidative stress-reduced expression of beta cell-fate markers such as duodenal homeobox factor-1 (Pdx-1), musculoaponeurotic fibrosarcoma oncogene homolog A (MafA), paired box 4 (Pax4) and homeobox protein Nkx6.1, decreases the expression of insulin (Ins) and glucose transporter GLUT1 and promotes the expression of progenitor markers such as SRY-box transcription factor 9 (SOX9) and hairy and enhancer of split-1 (HES1), suggesting occurrence of dedifferentiation process. By reducing expression of Pdx-1, oxidative stress may also lead to overexpression of alpha cell specific aristaless-related homeobox gene factor (Arx) and consequently to transdifferentiation of beta cells into alpha cells.

Numerous data indicate that oxidative stress and redox-related mechanisms stimulate de- or trans-differentiation processes in beta cells by interrupting the expression of beta cell maturity transcription factors (94), which has been illustrated in **Figure 2**. Initial studies demonstrated that prolonged culturing of insulin secreting HIT-T15 cells in high glucose concentration (11.1 mM) compromised insulin gene expression and GSIS resulting from loss of Pdx-1 and MafA binding to insulin promoter (95, 96). Those glucotoxicity effects were related to oxidative stress-induced posttranscriptional loss of Pdx-1 mRNA and posttranslational loss of MafA protein, which was further supported by findings showing that antioxidants such as N-acetylcysteine (NAC) or aminoguanidine improved insulin gene expression by preserving Pdx1 and MafA binding to insulin promoter in high glucose cultured HIT-T1 cells (97). Later on, it has been shown that H_2_O_2_ reduces insulin gene expression in rat islets by repressing binding of Pdx-1 to insulin promoter (98). Negative effects of ROS on Pdx-1 activity are probably mediated by JNK pathway and Foxo1 transcription factor (99). Namely, inhibition of JNK protects beta cells from ROS-mediated reduction of insulin expression, whereas activation of JNK induces nuclear translocation of Foxo1. In accordance, siRNA-inhibited expression of Foxo1 in H_2_O_2_-treated HIT-T15 beta cells keeps cytoplasmic localization of Foxo1 and nuclear localization of Pdx-1 along with insulin expression (100). Likewise, H_2_O_2_ provokes cytoplasmic localization of MafA and prevents its stimulating effect on insulin gene expression (101). Therefore, hyperglycemia-induced cytoplasmic translocation of MafA is considered to be an early indicator of beta cell dysfunction. Presented data strongly suggest that redox status affects subcellular localization of key insulin transcription factors as well as their regulators (11).

Leenders and coworkers (10) used human islets and human EndoC-βH1 cell line to investigate the effects of H_2_O_2_-induced oxidative stress on identity and function of human beta cells in diabetic conditions. They found that treatment with H_2_O_2_ promoted cell stress response and reduced expression of insulin and glucose transporter GLUT1 in human EndoC-βH1 cells associated with decreased expression of beta cell fate markers MafA, Pdx-1, paired box 4 (Pax4), Nkx6.1, as well as of beta cell-related genes Pax6, Nkx2.2, NeuroD1, Kir6.2, MafB and Foxa2. Simultaneously increased expression of progenitor cell-specific SOX9 and hairy and enhancer of split-1 (HES1) genes in H_2_O_2_-treated EndoC-βH1 cells suggests occurrence of dedifferentiation mechanisms (10). In accordance, usage of primary human islets and human islets transplanted into HFD fed mice revealed that widely used immunosuppressant in organ transplantation therapies, tacrolimus, accelerates development of diabetes by inducing loss of beta cell maturity features through metabolic stress-activated Foxo1 and reduced expression of MafA (102). Oxidative stress-related loss of beta cell identity is supported by findings showing that overexpression of Gpx1 or treatment with Gpx mimetic agents could restore expression of Pdx-1 and/or MafA in islets of diabetic mice (103, 104). At the same time, those findings strongly indicate that targeting of antioxidant mechanisms could play a significant role in preserving beta cell identity and function in diabetic condition.

## Natural antioxidants against beta cell failure

Current diabetes therapy includes the use of insulin and hypoglycemic drugs, as well as dietary correction. Various drugs exert their antidiabetogenic effect by stimulating insulin secretion, reducing the process of gluconeogenesis in the liver, increasing peripheral glucose absorption or delaying intestinal glucose absorption (105). Nevertheless, glycemic control is established in less than 50% of diabetic patients and results of up-to-date therapy are far from expected (106). Over time, patients become resistant to treatments, some drugs display side or even toxic effects, while insulin therapy carries the risk of life-threatening hypoglycemia (107). Such limitations in diabetes management have stimulated efforts to improve therapeutic approaches. One of the biggest challenges in this direction is certainly the recovery and preservation of functional beta cells. For that cause, several potential strategies emerged such as stimulation of existing beta cells to proliferate, islet transplantation from cadaveric donors, transplantation of beta cells generated *in vitro* from stem cells or cellular reprogramming of other types of pancreatic cells (50, 108). However, it turned out that stimulation of proliferation and transplantation are not quite effective or available, while potential of cellular reprogramming will be discussed in chapter 5. A major obstacle in using stimulated proliferation of beta cells in diabetes therapy is diminishment of the proliferative capacity of beta cells over time due to lower replication potential of human beta cells in comparison to rodents (109, 110). On the other hand, even when exceeding immune-mediated rejection, islet transplantation carries a great risk of progressive islet decline due to increased secretory demand, hyperglycemia, inflammation and oxidative stress (111). Besides, successful preservation and function of islet grafts is counteracted by oxidative stress in isolated islets which initiated the development of strategies, such as ROS reduction or induction of antioxidant enzymes, to reduce oxidative stress in islets before transplantation (112, 113).

Given the indisputable role of non-physiological oxidative stress in structural and functional disruption of beta cells, the use of antioxidants could have significant therapeutic effects. However, antioxidant supplementation with vitamins (A, C, E), coenzyme Q10 (CoQ10), enzymatic antioxidants-like mimics (CAT/GPx/SOD/mimetics), flavonoids, β-carotene, NAC, selenium and zinc in human clinical trials revealed limited beneficial effects probably due to their poor stability, solubility, permeability and specificity (114–116). For example, results from clinical trials regarding the beneficial effects of vitamins C and E in reducing various oxidative stress-related diseases are inconsistent due to almost an equal number of studies showing positive or no significant effect (116). This has stimulated efforts in finding efficient delivery systems to enhance the effects of antioxidant supplements in diabetes treatment as well as the examination of antidiabetogenic properties of plant-based preparations. Namely, herbal preparations contain complex combination of polyphenolic compounds that can act synergistically, antagonistically and additively against oxidative stress and different pathogenic mechanisms in diabetes (117). Coexistence of various antioxidant compounds in plants enables the maintenance of the so-called antioxidant chain - after the neutralization of free radicals, the antioxidant is recycled thanks to the next antioxidant in the chain (118). Therefore, usage of synthetic antioxidants cannot replace the consumption of food rich in antioxidants. Clinical trials such as phase II clinical trial (NCT02801448) with broccoli sprout extract in T2D patients (119), phase II/III clinical trial (NCT03262363) evaluating the effect of curcumin on antioxidant capacity and renal function in diabetic nephropathy (120), or phase II trial (NCT00811889) determining the effects of Nrf-2 activator bardoxolone methyl in T2D patients with chronic kidney disease (121) displayed promising results for the treatment of diabetic complications. These studies encourage further investigation of the beneficial effects of natural compounds in diabetic condition.

Sweet chestnut (*Castanea sativa* Mill.) is the most consumed among the twelve world chestnut species and is known for its antioxidant properties (122). Extracts prepared from three different parts of sweet chestnut (catkins, leaves and spiny burs) displayed high phenolic and flavonoid content and protected Rin-5F beta cells from STZ-induced oxidative stress and death by lowering DNA damage, lipid peroxidation and increasing GSH level (123). Positive effect of chestnut extract on beta cell viability against STZ toxicity was more pronounced in cooperation with extract prepared from edible mushroom *Lactarius deterrimus* (117). Combination of *C. Sativa* (spiny burs) and *L. deterrimus* extracts (MIX Ld/Cs) displayed high *in vitro* ROS-scavenging activity originating from chestnut extract and good NO-scavenging activity exhibited by mushroom extract. Moreover, MIX Ld/Cs acquired high ferrous (Fe^2+^) chelating effect despite the very low chelating activities of individual extracts. Analysis of underlying mechanisms revealed that MIX Ld/Cs exerted protective effect against STZ-induced beta cell death and improved their function by lowering GSH oxidation, NO-output and activity of NF-kB-p65 (117). When applied to STZ-diabetic rats at dose of 60 mg/kg (daily/4 weeks), *L. deterrimus* extract alleviated diabetes induced hyperglycemia and hyperlipidemia, lowered the level of glycated serum proteins and adjusted the diabetes-induced redox imbalance in the circulation (124). Increased activities of CAT and SOD enzymes in the circulation were accompanied by increased levels of glutathionylated proteins and free intracellular thiols. This systemic antioxidant effect was accompanied by restrained islet destruction and partially restored number of insulin-positive cells in diabetic animals through decreased level of advanced glycation end products (AGEs) and elevated expression of chemokine CXCL12 that mediated prosurvival pathway. Namely, analysis of the pro-survival protein kinase B (Akt) kinase and proliferating cell nuclear antigen (PCNA) indicated that mushroom extract shifted the balance from beta cell death in favor to survival and proliferation. Considering the presence of quiescent pancreatic cells with proliferative potential, described protective mechanisms of mushroom extract could be beneficial in initial stages of diabetes through regeneration of existing beta cell mass, supporting a therapeutic potential of *L. deterrimus* extract in diabetes treatment (124).

Traditional use of common centaury (*Centaurium erythraea* Rafn) for diabetes treatment in numerous Mediterranean countries stimulated *in vitro* and *in vivo* examination of its protective effects on pancreatic beta cells. Application of *C. erythraea* extract (200 mg/kg/day; 30 days) to STZ-diabetic rats recovered pancreatic islet morphology along with reduction of blood glucose level (125). Such beneficial effects of *C. erythraea* extract were attributed to its antioxidant properties and decreased oxidative damage of beta cells owing to increased level of GSH and stimulated activities of SOD, GPx and CAT enzymes in pancreas. Composition characterization of *C. erythraea* methanol extract by Đorđević et al. (8) revealed dominant presence of secoiridoids and abundant content of polyphenols such as phenolic acids, flavonoids and xanthones, while *in vitro* biochemical assays unveiled its strong reducing power, H_2_O_2_- and NO-scavenging activities. This is in line with data showing that *C. erythraea* extract reduced cell death by alleviating lipid peroxidation, protein S-glutathionylation, DNA damage and by correcting the expression and the activity of MnSOD, CuZnSOD, CAT, GPx and GR enzymes in STZ-treated Rin-5F beta cells (66). In parallel, *C. erythraea* extract modulated redox sensitive pathways mediated by NF-kB-p65, Nrf-2, forkhead box O3 (FOXO3A) and specificity protein 1 (Sp1) and promoted the activity of Pdx-1 and MafA proteins after STZ-trigered oxidative stress in Rin-5F cells. In addition, when applied to STZ-induced diabetic rats at a dose of 100 mg/kg, for 4 weeks, *C. erythraea* extract contributed to protection of beta cells in islets and improved the levels of activated pro-survival protein kinase B (p-Akt), insulin and its GLUT-2 receptor in pancreatic sections (66). *C. erythraea* extract also provided increased viability and insulin expression/secretion in Rin-5F cells after sodium nitroprusside (SNP)- or H_2_O_2_-induced redox imbalance (126). Unlike to H_2_O_2_ which directly causes oxidative damage and provides ^•^OH formation, SNP releases NO^•^ and induces cytotoxicity by nitrosative stress. SNP- and H_2_O_2_-promoted lowering of GSH/GSSG ratio, alterations in the activities of GPx, GR, CAT, MnSOD and CuZnSOD enzymes and the increase in DNA damage, protein S-glutathionylation and lipid peroxidation in Rin-5F beta cells was ameliorated by *C. erythraea* extract probably through its NO- and H_2_O_2_-scavenging activities (8, 126). This is especially important considering that NO^•^ and/or H_2_O_2_ overproduction negatively affects proliferation of beta cells as well as their ability to produce and secrete insulin (127). Underlying mechanisms of such detrimental NO^•^ and H_2_O_2_ effects are based on the post-translational modifications of Pdx1 and MafA factors crucial for insulin gene expression (11). It can be assumed that *C. erythraea* extract increased expression of insulin in SNP- and H_2_O_2_-treated beta cells by protecting Pdx1 and MafA from oxidative damage which is in correlation with *C. erythraea* extract-stimulated Pdx1 and MafA activity and expression of insulin in STZ-treated beta cells (66). Those findings are further supported with data showing that targeting of redox-induced modifications of regulatory factors such as kelch-like ECH-associated protein 1 (Keap1), protein kinase C (PKC), inhibitor of nuclear factor kappa-B kinase subunit beta (IKKβ), NF-kB, CCAAT/enhancer binding protein β (C/EBPβ), p38 and extracellular signal-regulated kinase (ERK) kinases or Mn-/CuZn-SOD and CAT enzymes are potential therapeutic strategies in treatment of diabetes pathogenesis (8, 105, 114, 128).

**Figure 3:**
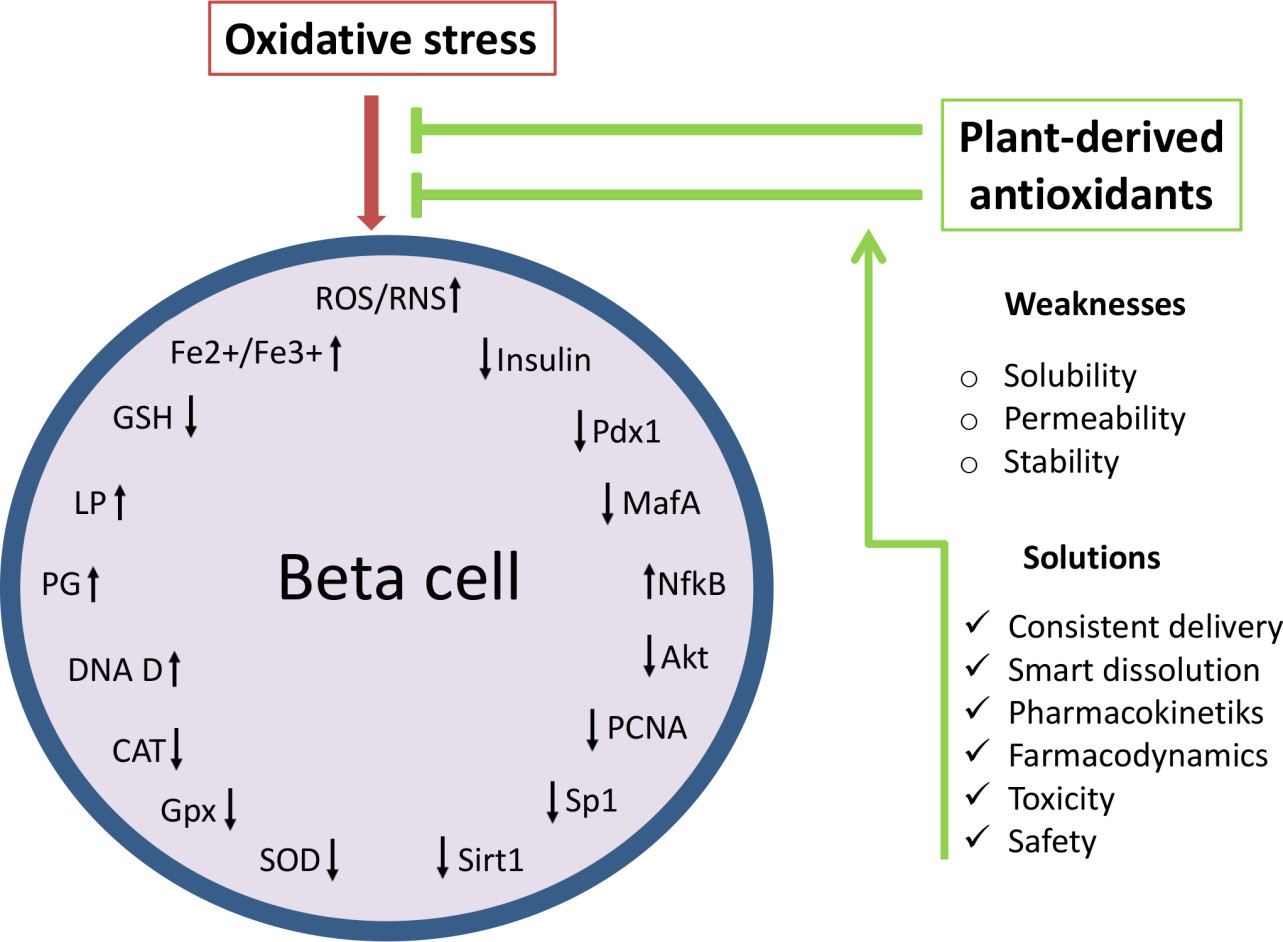
Effects of natural antioxidants (polyphenolic compounds) on oxidative stress-related targets: ROS production, redox modifications and ROS-induced injury of beta cells. Oxidative stress reduces the functioning and survival of beta cells through oxidative damage and modifications of macromolecules, disruption of the antioxidant defense system and reduction of beta cell maturity genes. Thus control of oxidative stress by plant-derived preparations has a great potential in the treatment of diabetes. However, the protective effect of natural antioxidants on beta cells can be enhanced by the improvement of a current antioxidant delivery approaches. ROS/ROS, reactive oxygen and nitrogen species; Fe^2+^/Fe^3+^, ferrous ions; LP, lipid peroxidation; PG, protein glycosylation; DNA D, DNA damage; SOD, superoxide dismutase; CAT, catalase; GPx, glutathione peroxidase; GSH, glutathione; NF-kB, nuclear factor kappa B; Akt, protein kinase B; Pdx1, duodenal homeobox factor-1; MafA, musculoaponeurotic fibrosarcoma oncogene homolog A; PCNA, proliferating cell nuclear antigen; Sp1, specificity protein 1; Sirt1, Silent information regulator 1.

As not being initial factor in diabetes development and progression but rather being induced by immune reaction, inflammation and glucolipotoxicity, targeting of oxidative stress at present is seen as ancillary treatment in diabetes therapy (27). However, numerous studies dedicated to illumination of the mechanisms involved in beta cell failure strongly suggest that control of oxidative stress has a great potential in the treatment of diabetes. The main disadvantage of effective use of antioxidants such as polyphenolic compounds in diabetes therapy seems to be their insufficient stability, permeability and aqueous solubility in conventional application. Phenolic compounds cannot be absorbed in their native form and are subjected to enzymatic hydrolysis in the small intestine (around 48% of all polyphenols) and large intestine (about 42%) remaining only 10% in undigested and intact form (129). Thus, the effective concentration that reaches target tissue and displays biological effect is far less from the applied dose of phytoconstituents (130). For that cause it is demanding to develop antioxidant delivery approaches with consistent delivery and smart dissolution that could precisely target ROS production, redox modifications as well as ROS-induced injury and death of beta cells (115, 131). Since normal functioning of beta cells requires balanced ROS production, novel antioxidant delivery systems also have to provide adequate modulation of antioxidant defense to elicit beneficial not adverse effects. Advantages and weaknesses of beta cell protection by natural antioxidants are presented in **Figure 3**.

## Oxidative stress-related epigenetic regulatory mechanisms as a therapeutic target for diabetes

Developmental studies have provided evidence that pancreas maintains a certain level of cellular plasticity under pathological conditions (132). This implies that under given environmental conditions beta cells can undergo dedifferentiation and transdifferentiation, with ability of re-differentiation depending on severity and duration of stressors (50). By avoiding prolonged exposure to those insults it would be possible to recover mature identity of pre-existing beta cells and their normal functioning. In accordance, advances in experimental and clinical studies have illuminated the substantial role of epigenetic regulatory mechanisms in the development and function of beta cells in physiological conditions, as well as in triggering islet autoimmunity and/or beta cell dysfunction and decline in both T1D and T2D (21, 133).

Epigenetic mechanisms regulate complex interplay between environmental or other external factors and gene expression (134). Epigenetic modifications are highly dynamic and include DNA methylation/demethylation, histone posttranslational modifications and non-coding microRNA (miRNA) expression that all influence chromatin organization and gene regulation without changing DNA sequence. A load of scientific publications supports a fundamental principle that the specific combination of epigenetic modifications is necessary for the identity of each pancreatic cell lineage. Any dysregulation of pancreatic cell’ epigenetic signatures are a key event in the pathogenesis of diabetes and therefore the molecular machinery that defines these epigenetic signatures can be potential targets in the therapeutic development of diabetic epidrugs (21).

### Oxidative stress and epigenetic modifications in diabetes

Elevated glucose level in diabetic condition affects biochemical and metabolic pathways which is also reflected on epigenetic modifications (134). Reduced level of a methyl group donor SAM in diabetes may interrupt methyltransferase activity and inhibit methylation of DNA and histone proteins (135, 136). The evaluation of the histone modification role in T1D cohort revealed association between levels of glycohemoglobin and H3K9 acetylation in the monocytes, as well as altered DNA methylation of 3′ UTR of thioredoxin-interacting protein, suggesting its potential use as a biomarker for oxidative stress (137, 138). In study of elderly T2D patients, reduced level of DNA methyltransferases (DNMTs) in leucocytes was recorded (139). T2D pathogenesis is considered to be associated with alterations in DNA methylation due to rise in level of short-chain fatty acids, which lead to hypomethylation and dysregulation of pro-inflammatory cytokine genes (140). In accordance, decreased levels of Suv39H1 methytransferase and trimethylated lysine 9 residue of histone 3 (H3K9me3) at interleukin-6 promoter were detected in vascular smooth muscle cells of T2D mice (141). Diabetes pathogenesis is also associated with alterations in miRNA levels, for example miR-21 and miR-210 are taken as T1D biomarkers, while lowering of miR-126 is connected with T2D development (142, 143).

Oxidative stress significantly contributes to the control of epigenetic regulatory mechanisms (144). Oxidative stress-induced DNA strand breaks and damage can alter DNA-protein and protein-protein interactions, thus leading to abnormal DNA methylation pattern (145). Oxidation of guanine within CpG islands produces 8-OHdG which stimulates DNA methylation of adjacent cytosine and reduces affinity of methyl-domain binding proteins. DNA methylation can be also stimulated by reactive molecules through cytosine deprotonation and accelerated interaction between DNA and SAM donor (146). Oxidative stress promotes DNMT1 and histone deacetylase (HDAC) 1 association and inhibits activity of HDACs such of Silent information regulator 1 (Sirt1) (147). In addition, multiple lysine residues on histone domains are also susceptible to harmful effects of oxidative stress (148). These changes in epigenetic modifications certainly lead to disturbances in the regulation of expression and activity of antioxidant enzymes and their regulators, which in turn promotes oxidative stress conditions. SOD gene expression is suppressed by increased DNA methylation, H4K20 methylation and H3K9 acetylation in diabetic conditions (149, 150). Nrf2, a central regulator of antioxidant defense genes and its regulator Keap1 are both epigenetically regulated by miRNAs and histone modifications (134).

### Epigenetic modifications and beta cell function

Epigenetic modulatory mechanisms are involved in the regulation of beta cell differentiation, proliferation, homeostasis and pathogenesis (21, 144, 151). Dhawan et al. (152) have demonstrated that beta cell identity relays on DNA methylation-mediated repression of Arx. Namely, lack of DNMT1 gene in transgenic mice led to trans-differentiation of beta cells into alpha cells, which was in correlation with Arx1 gene hypomethylation. DNMT1 deletion did not display large-scale alterations in gene methylation suggesting not just localized effect of DNMT1 activity, but also the crucial effect of epigenetic mechanisms in beta cell differentiation. Besides DNA methylation, histone acetylation has been demonstrated to be involved in the regulation of islet development and functioning and T2D pathogenesis (153). Application of HDAC inhibitors (HDACi) during rat embryonic development increased Neurogenin3 positive endocrine progenitor cells revealing a key role of histone acetylation in lineage specification of pancreatic alpha, beta, delta and PP cells (154, 155). In line with those data, HDACs were shown to be essential in diabetes pathogenesis since specific HDACi augmented beta cell mass and insulin secretion (156, 157). It has been shown that insulin expression is inversely correlated with CpG methylation level, i.e. insulin promoter is hypomethylated in beta cells and hypermethylated in other cell types (158). Accordingly, specific methylation profile of the proximal promoter of insulin gene was found to be associated with T1D (159). Therefore, differential methylation of DNA from insulin gene in circulation could be used as a biomarker of beta cell loss in T1D (160). Similarly, DNA methylation profiling of human islets from control and T2D donors revealed differential DNA methylation of 853 genes which can influence insulin secretion (161). These findings indicate important role of DNA methylation in insulin secretion and beta cell dysfunction in T2D. Moreover, in cooperation with histone acetyltransferase p300, Pdx1 enhances insulin expression under high glucose concentration by stimulation of chromatin opening near insulin promoter, while at low glucose levels Pdx1 inhibits insulin expression by recruitment of HDAC1 and HDAC2 (162, 163). Consistently, it was found that proteasomal degradation of p300 contributes to beta cell apoptosis in diabetes condition (164), while removal of Set7/9 methyltransferase and disturbance of active H3K4 histone mark in beta cells reduces Pdx1 expression and insulin secretion (165, 166). Numerous studies revealed the impact of miRNA regulation in beta cell failure. Among great number of miRNA, specific miR-375 was detected exclusively in pancreatic islets where it regulates beta cell programming, proliferation and glucose homeostasis (167). Targeted inhibition of miR-375 in mice disturbed islet morphology with the reduction in alpha and beta cell mass and impaired insulin secretion (168, 169). IL-1β/TNF-α-induced damage of MIN6 cells, islets of NOD mice and human islets, was mediated by miR-21, miR-34a and miR-146a overexpression (170), while increased expression of miRNA-29a/b/c in islets of pre-diabetic NOD mice was associated with impaired GSIS and induction of apoptosis (171). In newly diagnosed T1D children, miR-25 was found to be associated with glycemic control and function of residual beta cells (172).

It is evident that epigenetic regulation plays an essential role of in the development, viability and function of beta cells, as well as, that diabetic environment and oxidative stress interfere with those epigenetic marks causing dysfunction and loss of beta cells. On the other hand, plasticity of epigenetic mechanisms strongly encourages the development of a novel diabetic therapeutic strategies based on reversing epigenetic modifications in order to enable the recovery of beta cells.

### Epigenetic targets in diabetes therapy and potential use of natural antioxidants

Association between epigenetic mechanisms and diabetes pathogenesis has stimulated development of new therapeutic strategies with ability to reverse epigenetic modifications, such as epidrugs and epigenome editing (133, 173). In accordance, synthetic compounds have been intensively studied as potential epigenetic modifiers primarily in cancer, but also in other pathological states, including diabetes. DNMTs inhibitors such as 5-azacytidine (Aza, Vidaza) and its derivative 5-aza-2′-deoxycytidine (DAC, Decitabine) are currently approved drugs for the treatment of hematological malignancies in elderly patients, but were also shown to be effective in the treatment of inflammatory diseases (174–176). Treatment of non-obese diabetic (NOD) mice with DAC prevented diabetes development by cyclophosphamide, while application of DAC to HFD fed ob/ob mice improved insulin sensitivity (177, 178). HDAC inhibitors valproic acid and trichostatin A reduced apoptosis and improved proliferation and function of beta cells in T1D animals (179, 180). Acting as agonists of HDAC enzyme Sirt1, medications such as metformin and fenofibrate stimulated insulin secretion and improved glucose metabolism in T1D mice (181, 182).

**Figure 4:**
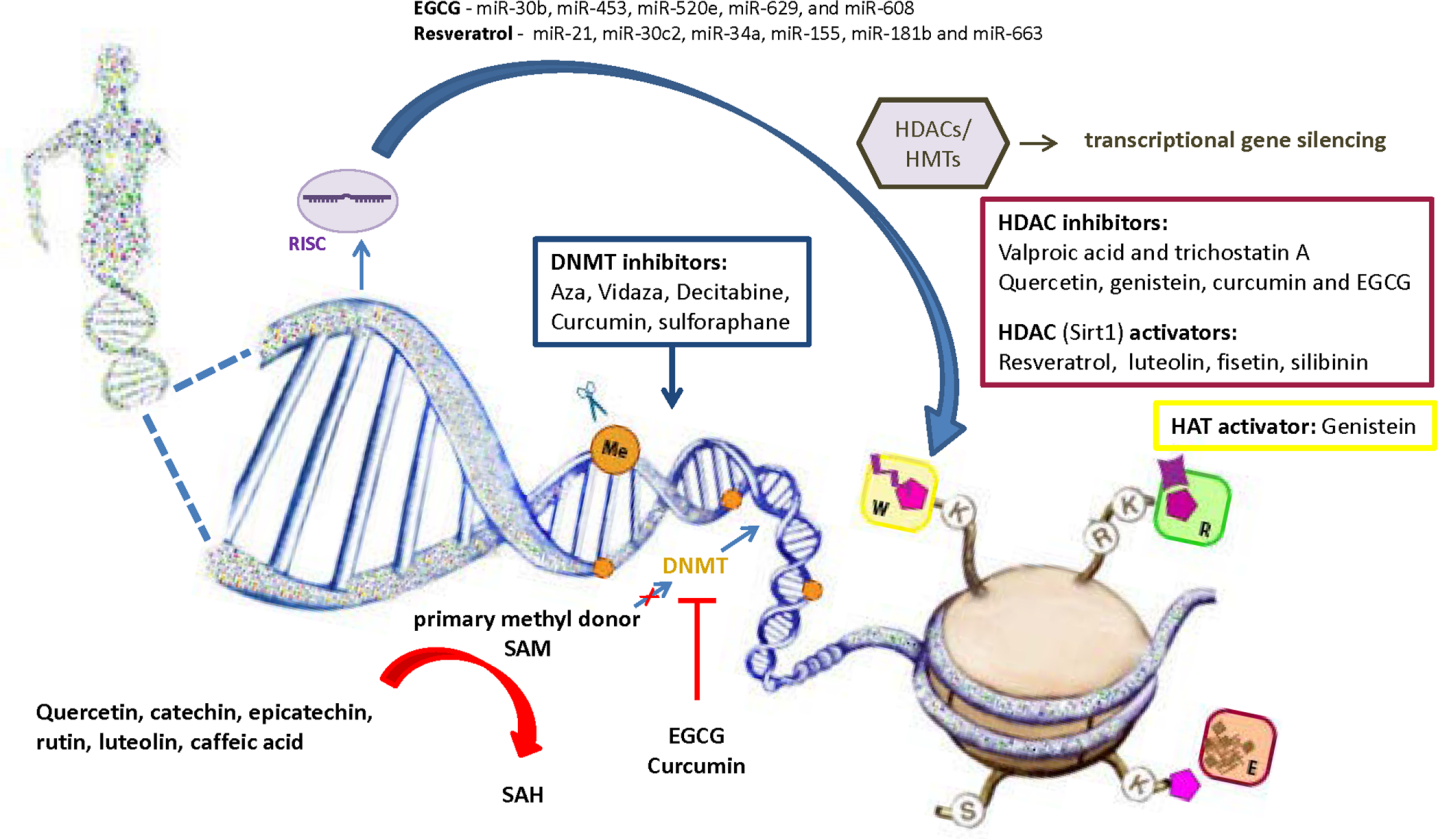
Role of natural antioxidants in the regulation of epigenetic mechanisms involved in diabetes pathogenesis. Antioxidants are becoming powerful alternative approach in epigenetic therapy. Besides synthetic compounds as a potential epigenetic modifiers (Aza, Vidaza, Decitabine (DAC), valproic acid and trichostatin A), it has been shown that polyphenolic compounds have the ability to modulate epigenetic related mechanisms including DNA methylation, histone modification and regulation of miRNA expression. SAM, S-Adenosyl methionine; SAH, S-adenosylhomocysteine; DNMTs, DNA methyltransferases; HATs, Histone acetyltransferases; HDACs, Histone deacetylases; HMTs, Histone methyltransferases; RISC, RNA-induced silencing complex; EGCG, epigallocatechin gallate; W, writers of histone marks; R, readers of histone marks; E, erasers of histone marks.

Considering the role of oxidative stress in diabetes development *via* affecting epigenetic mechanisms, the use of antioxidants becomes powerful alternative approach in epigenetic therapy. In addition to the confirmed anti-inflammatory and antioxidant activity of polyphenolic compounds, their effect on epigenetic regulation is increasingly being recognized (**Figure 4**). It has been shown that polyphenolic compounds have the ability to modulate epigenetic related mechanisms including DNA methylation, histone modification and miRNA level (183). Despite evident involvement of epigenetic regulatory mechanisms in a number of diseases, the majority of studies investigated the effects of natural polyphenols in the treatment of cancer, and these data are certainly valuable guidelines for research in other fields. So far, only limited number of studies has reported causative relationship between dietary polyphenolic compounds and epigenetic modifications in diabetes pathogenesis. However, it is undeniable that dietary deficiency or excesses in methyl donors could affect global changes in DNA and/or histone methylation patterns (144). The regeneration of primary methyl donor SAM from S-adenosyl homocysteine (SAH) also depends upon the presence of certain nutrients such as zinc, folate, choline, betaine or vitamins B2, B6 and B12 that serve as cofactors or intermediates (135, 184). Given that SAH has a negative regulatory effect on methyltransferase activity, its cellular accumulation could inhibit methylation of DNA or histone proteins (144). Polyphenols may directly inhibit DNMTs by reducing their expression and activity or indirectly by lowering SAM levels. Epigallocatechin-3-gallate (EGCG) and curcumin inhibit DNMT1 through a direct interaction with enzyme (184, 185), while catechol-containing polyphenols such as quercetin, catechin, epicatechin, rutin, luteolin and caffeic acid act as a non-competitive DNMTs inhibitors by causing SAH overproduction due to usage of SAM as a methyl donor for their own methylation (183). Regarding the histone mark modifications by polyphenols, it has been found that resveratrol-stimulated histone deacetylation through activation of Sirt1 resulted with improved glucose level and increased insulin secretion in experimental animals (186). Natural compounds such as quercetin, genistein, curcumin and EGCG were identified as HDACi (187). EGCG corrects gene expression of tumor suppressor genes by inhibition of HDAC activity and stimulation of H3K9/H3K14 and H4K5/H4K12/H4K16 histone acetylation in cancer cells (188). Genistein also can act as histone acetyltransferase (HAT) activator and Sirt1 inhibitor, while EGCG and curcumin display HAT inhibitory activity (189, 190). EGCG decreases the level of miR-30b, miR-453, miR-520e, miR-629, and miR-608 in human hepg2 cells (191), and resveratrol regulates the activity of inflammation-related miRNAs (miR-21, miR-30c2, miR-34a, miR-155, miR-181b and miR-663) in T2D patients (192).

Phenolic phytochemicals may also regulate gene expression of antioxidant enzymes through alteration of epigenetic modifications (51). This could be achieved by epigenetic activation of Nrf-2 transactivator of antioxidant genes, as it was reported for curcumin, sulforaphane, quercetin and reserpine (193). Curcumin and sulforaphane were identified to increase expression of Nrf2 by demethylating its promoter and decreasing DNMT1 enzyme (194, 195). Luteolin was also reported to activate Nrf-2 gen in heme oxygenase-1 induction and to enhance HDACs and repress HATs in high glucose-treated THP-1 cells (196, 197). Besides, combination of luteolin with fisetin reduces ROS formation and promotes Sirt1expression (198). Sirt-1 plays a multiple roles in beta cells because it is considered to be a strong stimulator of GSIS process and important regulator of physiological level of autophagy (199, 200). It has been shown that a polyphenolic flavonoid silibinin reduced apoptosis of beta cells in STZ-diabetic mice by inducing Sirt-1 expression and by recovering physiological autophagy (200). Despite growing body of evidence, many pieces of diabetes-oxidative stress-epigenetic puzzle are still missing and require further investigation. A number of issues in targeting epigenetic mechanisms by drugs or natural epigenetic modifiers have to be clarified including their adequate dosage, pharmacokinetics, pharmacodynamics, toxicity and safety (134). This is especially important considering the multi-factorial nature of diabetes which requires multitargeted approach for its prevention and treatment.

### CRISPR/Cas9 epigenome editing in attenuation of diabetes

Prokaryotic clustered regularly interspaced short palindromic repeat (CRISPR)/Cas systems were repurposed to be used as a tool for modulation of gene expression by the spatial positioning of genomic loci, genome and epigenome editing (201). The most attractive gene editing tool is the class 2 CRISPR/Cas9 system, in which a single Cas9 protein from *Streptococcus pyogenes* (SpCas9) targets specific DNA sequences (202). This system is composed of a single-stranded guide RNA (sgRNA) usually 20 bp long and Cas9 endonuclease. The designed unique sgRNA, by recognizing complementary bases of the target site, directs Cas9 to the targeted DNA containing upstream 2-5 bp DNA sequence known as protospacer adjacent motif (PAM) (usually 5’-NGG-3’; N - any nucleotide base), compatible to Cas9 protein (203). Positioning of Cas9 to a target DNA site initiates the formation of an RNA-DNA hybrid and Cas9 mediated DNA cutting into double strand breaks (204). Thus, by inducing repair machinery (non-homologous end joining (NHEJ) or homology directed repair (HDR)), CRISPR/Cas9 system provides targeted genome modifications of DNA, including insertions and deletions (205).

The CRISPR/Cas9 platform has a multiple advantages in genome and epigenome editing in comparison to the other editing technologies such as transcription activator-like effector nucleases (TALENs) or zinc-finger nucleases (ZFNs) (206). Concisely, TALEN or ZFN platforms imply *de novo* nuclease synthesis for each target sequence and are more demanding in terms of time and costs. On the other hand, Cas9 enzyme does not require reengineering because its DNA sequence targeting relies on sgRNA alterations (207). Moreover, CRISPR/Cas9 system enables simultaneous editing of multiple target loci, making this technology more comfortable and powerful therapeutic approach for the treatment of diseases.

Several gene editing attempts that relay on CRISPR/Cas9 targeted gene activation tools have been developed in order to modify expression of certain genes in primary human pancreatic islet cells (208). Bevacqua and colleagues (208), using CRISPR/Cas9 targeting of PDX1 in primary human islets showed loss-of-function phenotypes, as well as using dCas9 fused to the activation domains VP64-p65-Rta23, manage to activate gene expression for endogenous genomic sequences of human islet cells, underlining an essential function of PDX1 in primary mature human islet beta cells. Previously, CRISPR-based targeting has been reported in human stem cell-derived insulin-producing cells (209) and in immortalized beta cell lines, EndoCβH3 (210) which unfortunately represent surrogate cell types that are genetically and biologically far from genuine pancreatic islet cells. To overcome these problems, researchers in the field of diabetes decided to use CRIPSR/Cas9 gene editing of diabetic patient-derived iPSCs and to induce their differentiation to autologous pancreatic beta cells. These cells reversed pre-existing STZ-induced diabetes when transplanted into mice (211). In addition, similar discovery, so called VCTX210, resulted in the Phase 1 clinical trial. The VCTX210, is an allogeneic, gene-edited, stem cell-derived therapy designed as a best-in-class treatment for T1D and insulin-dependent T2D. The therapy is designed to replace the pancreatic beta cells that are lost in diabetes, and is developed using a CRISPR/Cas9 gene-editing approach on CyT49 human pluripotent stem cells engineered to avoid destruction by patient’s autoimmune attack (212).

**Figure 5:**
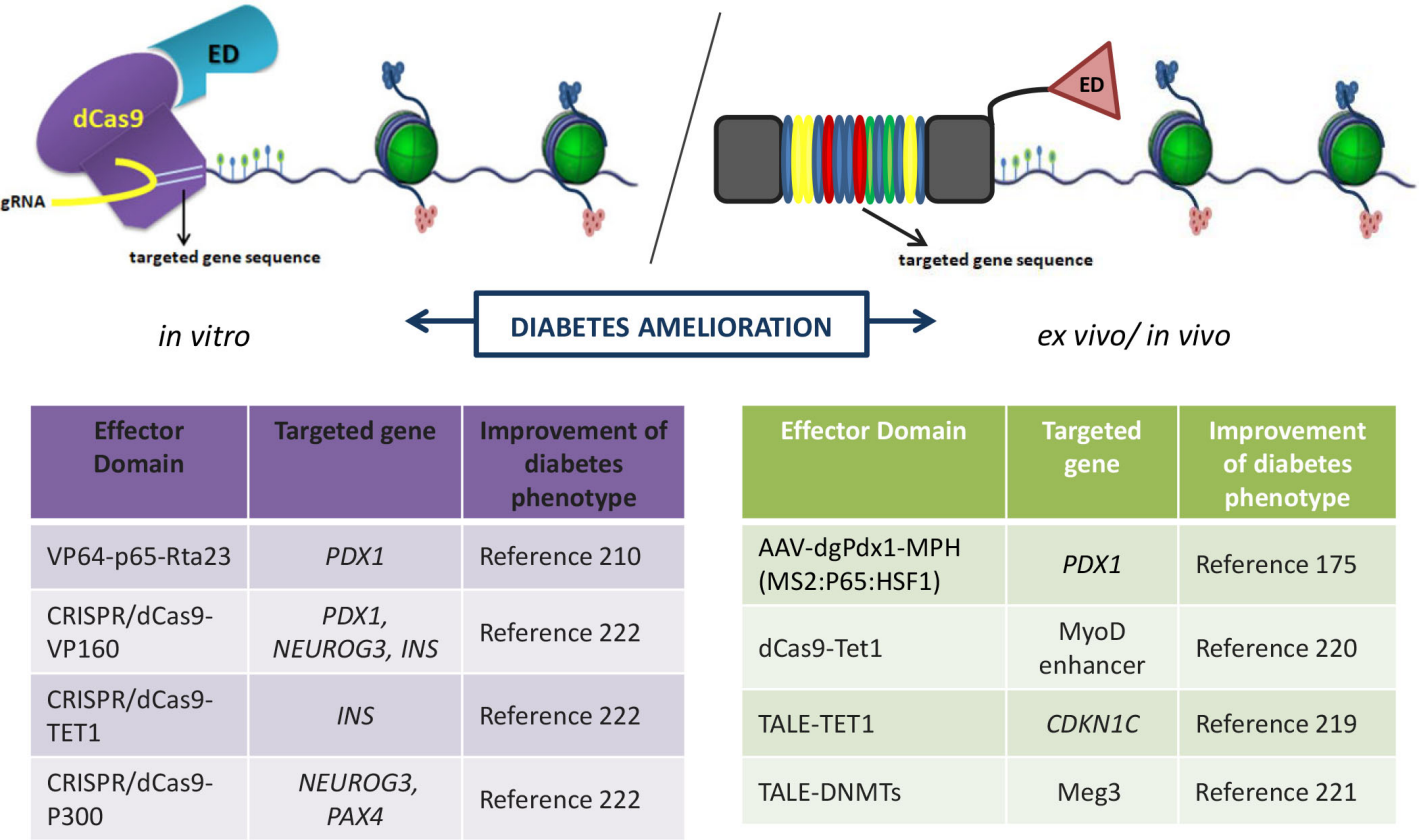
Improvement of diabetic conditions by epigenome editing using CRISPR/dCas9 synthetic tools. The epigenome editing is cutting edge research tool for deciphering the pathology of diabetes and for offering a potential cure. The existing epigenetic editing tools are consisting of target DNA sequence recognition part (TALEN or dCas9) and linked to different effector domains (ED). ED could be any of epigenetic modifiers of DNA (DNMTs and TETs) or histones (histone modifying enzymes). In regard of diabetes treatment, several *in vitro* and *in vivo* studies based on usage of these tools have been recently released and are listed in the inset tables. gRNA-guide RNA.

The epigenome editing emerged as another promising research option for deciphering the pathology of diabetes and for offering a potential cure (**Figure 5**). Epigenome editing may have certain advantages over genome editing given that it affects reversible epigenetic mechanisms of genome regulation. Besides, epidrug-based epigenetic therapies could have more pronounced off-target effects than methods for targeted epigenome editing such as CRISPR/dCas9 methodology. Since creation of DNA double-strand breaks in genome-editing systems limits their clinical use due to potential creation of unwanted mutations with harmful effects, the CRISPR/Cas9 system had to be redesigned in order to drive a targeted regulation of endogenous gene expression without creating DNA double strand breaks. To repurpose the CRISPR/Cas9 for genome regulation instead of genome editing, a catalytically inactive version of Cas9 (dCas9) has been created as a platform for RNA-guided transcription regulation (CRISPRi). In this scenario, CRISPR/dCas9 was targeted to the protein-coding region of targeted genes and by forming RNA-DNA adducts, it blocks RNA polymerase and transcript elongation (213). Despite the objective difficulties accompanying the *in vivo* implementation of this editing system, Liao and coworkers (173) succeeded to activate endogenous target genes in mice using CRISPR/Cas9-mediated target gene activation (TGA) tool. Overexpression of Pdx1 in liver cells by CRISPR/Cas9-mediated TGA system (used with dgRNA that deactivates Cas9) led to their transdifferentiation into insulin-secreting cells in a mouse STZ-induced T1D model of diabetes (173). This effect was followed up by a partially reduced STZ-induced hyperglycemia along with increased serum levels of insulin in STZ-diabetic mice. The specificity of this robust system is that it provides the trans-epigenetic remodeling of histone marks through dgRNAs-mediated recruitment of Cas9 and transcriptional machinery to target sites. Those findings strongly suggest that CRISPR/Cas9/dCas9 TGA tool may provide engineering of cell fate in order to produce cell types necessary to restore particular physiological functions *in vivo*.

The blooming in the field of epigenetic editing started with several constructs design to allow targeted editing of specific epigenetic marks to alter the expression of specific genes (214–216) (**Figure 5**). The epigenetic editing is based on tools that consists of target DNA sequence recognition part (TALEN, ZNF or dCas9) linked to different epigenetic modifiers of DNA (DNMTs and TETs) or histones (histone modifying enzymes). Recently, few studies based on usage of these tools in diabetes treatment have been released. Ou et al. (217) used the TALE-TET1 system for demethylation of the imprinted control region 2 (ICR2) which resulted in decreased expression of CDKN1C and increased replication of pancreatic beta cells. Also, targeted demethylation of the distal MyoD enhancer by dCas9-Tet1 and fibroblasts reprogramming to myoblasts has been shown (218). Moreover, using this approach, Kameswaran et al. (219) identified an intronic enhancer that seems to regulate allele-specific expression at the imprinted DLK1-MEG3 locus which is dysregulated in islets from T2D patients (219). Furthermore, the utility of CRISPR tools and multiplexing (targeting multiple genes in the same time) for epigenetic editing and directed cellular differentiation of pancreatic beta cells have been recently shown (220). In this paper, authors used the CRISPR/dCas9-VP160, CRISPR/dCas9-TET1 and CRISPR/dCas9-P300 systems for multiplex epigenetic editing and activation of human pancreatic beta cell genes (*PDX1, NEUROG3, PAX4* and *INS*) essential for maintaining beta cell identity (**Figure 5**).

## Conclusion

The loss of pancreatic beta cell mass and function represents a central event in T1D and T2D pathogenesis despite differences in their development and progression. In order to emphasize the significance of targeting the redox-related mechanisms in diabetes management, this review has evaluated different aspects of the involvement of oxidative stress in the structural and functional disruption of pancreatic beta cells. Both, experimental and clinical research unambiguously highlights the adverse effects of oxidative stress on beta cell identity and viability through intermediation in various pathological mechanisms involved in beta cell malfunction, de-/trans-differentiation and death. Detrimental effects of oxidative stress on beta cells can be to accomplish either directly through oxidative damage of macromolecules, or indirectly by disturbances of numerous regulatory pathways including epigenetic mechanisms. Among other regulatory processes, identity of beta cell relays on DNA methylation-mediated repression of *Arx*, while insulin expression inversely correlates with DNA methylation level of insulin promoter. Accordingly, clinical studies provided evidence that T1D and T2D are associated with altered methylation profile of the insulin gene promoter indicating a key role of DNA methylation in insulin secretion and beta cell dysfunction. Oxidative stress-induced damage of DNA and proteins in diabetes can lead to abnormal DNA methylation pattern and histone modifications, which opens the avenue for the development of a new therapeutic approach based on reversing epigenetic modifications by targeting oxidative stress. This is supported by the experimental data showing that the correction of ROS production and modulation of redox-related signaling and modifications by plant derived antioxidants (such as polyphenolic compounds) can stimulate pro-survival pathways in beta cells under diabetic condition. Moreover, the ability of polyphenolic compounds to modulate epigenetic related mechanisms such as DNA methylation, histone modifications and miRNA level further encourages the use of antioxidants in epigenetic therapy. At least, this approach may provide the preservation of beta cells in early phases of diabetes or renewal of beta cell mass in the later stages of the disease.

To conclude, possibly there are several avenues where epigenetics may help in improving the diabetic conditions. Since differentially methylated insulin gene is recently detected as biomarker of progressive beta cell loss, the discovery of novel blood-based epigenetic biomarkers may be used to predict risk for diabetes development and development of its related complications. Furthermore, discovery of new epidrugs to serve as activators or repressors of different epigenetic enzymes relevant for DNA or histone modifications and regulation of miRNA expression may be potential future therapy for diabetes. Lastly, epigenetic editing and valuable advantages of CRISPR/dCas9 methodology, pave the way for the development of targeted epigenetic therapies against wide range of injuries and human diseases, not just diabetes (173). In addition, challenges in its implementation cannot be ignored. Choosing of unique Cas9 target sequences without homology to other genome regions and the improvement of structure and composition of gRNA will reduce off-target effects (221, 222). Finally, editing of DNA with irreversible permanent change of genome information carries unavoidable security risks and ethical problems (207). Likewise, a number of issues in targeting epigenetic mechanisms by natural and synthetic epigenetic modifiers have to be clarified including their adequate dosage, pharmacokinetics, pharmacodynamics, toxicity and safety. This is especially important considering the multi-factorial nature of diabetes which requires multitargeted approach for its prevention and treatment.

## Author contributions

Conceptualization: systematic literature search and writing, SD and MV. Review and editing: JAJ, AU, MM, NG, AT, JR, and MĐ. Supervision: MV. All authors contributed to the article and approved the submitted version.

## Funding

The Ministry of Education, Science and Technological Development of the Republic of Serbia supported this work (Grant No. 451-03-68/2022-14/200007).

## Conflict of interest

The authors declare that the research was conducted in the absence of any commercial or financial relationships that could be construed as a potential conflict of interest.

## Publisher’s note

All claims expressed in this article are solely those of the authors and do not necessarily represent those of their affiliated organizations, or those of the publisher, the editors and the reviewers. Any product that may be evaluated in this article, or claim that may be made by its manufacturer, is not guaranteed or endorsed by the publisher.
